# Nursing Sore Mouth or Puerperal Anæmia

**Published:** 1855-09

**Authors:** M. L. Knapp


### DEPARTMENT OF SELECTIONS.

### "NURSING SORE MOUTH," OR "PUERPERAL ANÆMIA."

BY M. L. KNAPP, M. D.

[CONCLUDED.]

*Remarks*—Our circular was sent to Dr. Judkins, understanding through some of his patients, whom he had treated for this affection, that he possessed skill in its treatment, and enjoyed some reputation for its successful management, beyond that of the generality of physicians. His success, it now appears, depends on the liberal use of *a salt of soda*, *tonics and astringents*, *with good food*, *and tepid ablutions daily* ; a course well calculated to promote, coax, urge, even *force* the nutritive process. This practice appears very rational, certainly, and in the absence of a true pathology, and the real cause of the affection not known, must be regarded as happy. When we shall have unfolded the *essential nature* of this anomalous affection, the why and wherefore of the success of Dr. Judkins' practice will be clear.

The points of particular interest, then, in Dr. Judkins' paper are, the apparent increase of this affection of late years—delicate ladies its subjects—its liability to recur—good, rich diet a preventive—pain and tormina of the bowels always present in marked cases, together with a watery diarrhœa, and *white flocculi floating in the dejections*, *as in cholera*. No other contributor to the literature of this affection, we believe, has taken notice of this last circumstance—a very important fact. Doubtless these floating floculi are cast-off patches or sloughs of the epithilium, the same as in cholera, and not mucous secretions, as Dr. Judkins supposes. The tendency to grave local lesions, and the development of tuberculosis, hemoptysis, etc., is another point of importance to be borne in mind in this very practical contribution.

Let us now consider the literature of this disease.

### LITERATURE OF NURSING SORE MOUTH.

The main contributions upon this supposed new form of disease, are from Drs. Hale, Backus, Channing, Bell, Wood, Shanks, Taylor, Holt, Ware, M'Gugin and King, in the United States, who have described it as it appeared in the extreme eastern, western, northern and southern States. These contributors have described the affection from personal observation, with the exception of Drs. Wood and Bell of Philadelphia, who have given it place in their standard systems of practice as a new form of disease on the testimony of others, and chiefly Drs. Hale and Backus, who were the first to call attention to this anomalous affection through the Medical Journals—Dr. Hale's article having appeared in 1830, and that of Dr. Backus in 1841.

Beside the above-named American contributors to the literature of this anomalous affection, we find that Dr. Marshall Hall, of London, wrote a treatise on a similar anomalous disease, in 1820, designated by the very indefinite appellation of "*a serious affection*," which, appearing to us the same as the nursing sore mouth of this country, we shall begin our synopsis by analysing :—

*Dr. Marshall Hall's Treatise.*—A review of this may be seen in the London Medical and Physical Journal for July, 1820, with copious extracts—our source of information. Dr. Hall appears to have encountered an epidemic of this affection in the early period of his career, and without venturing an opinion as to what it was, wrote a treatise of ninety-six pages on it, "*Cases of a serious affection chiefly occurring after delivery, miscarriage, etc., and of a similar affection unconnected with the puerperal state.*" The general character of the serious affection of Dr. Hall appears to us to be the same as that of puerperal anæmia, or the nursing sore mouth, since described in the Journals of this country—its victims are the same, and there is a striking similarity in the history and progress of the cases—the whole phenomena in fact, even to the mode of death. Dr. Hall's cases do not, perhaps, appear to have been characterized by as marked a tendency to ulceration of the mouth as the general run of cases described in this country, though, from the fact that those persons were among its frequent victims, who suffered from "aphthæ with irritable stomach," it is evident that sore mouth was a symptom generally in Dr. Hall's cases.

*The causes* of the affection Dr. Hall supposes to be, irritability and exhaustion following the shock, drain or fatigue of the system incident to parturition, abortion, or lactation.

*The subjects* of the disease are those exhausted by diarrhœa or other sickness previous to delivery; those of pale, icterode complexion, who had been anasarcous, or who had suffered from aphthæ with irritable stomach and bowels; those exhausted by repeated and prolonged uterine or other hemorrhage, or depletion for subduing inflammatory diseases; and the naturally delicate and feeble. He has seen it suddenly developed after venesection, and also after full purgation. Anxiety, alarm, and disturbance of mind have seemed to cause it. The affection in some instances came on in the latter period of pregnancy. Sudden and unexpected death sometimes occurred after delivery, or even after blood-letting. By a removal of the exciting cause, as a prolonged menorrhagia or an exhausting lactation, the prominent or urgent symptoms frequently ceased. The affection sometimes proved fatal after a more or less urgent, protracted, and varied course; and in other cases there was long-continued indisposition. The doctor attempts to arrange his cases under six varieties, as follow: "1. The acute; 2. The more continued; 3. With general symptoms; 4. With some predominant local affection; 5. As the effect chiefly of intestinal irritation; or 6. Of hemorrhage. The greater number of cases, however, do not admit of being referred to any one of these divisions, distinctly or exclusively, but assume a mixed character." He arranges his account of the symptoms with reference to the regions of the body affected, as follows:

*The head.*—"Severe pain, beating and throbbing, rushing or cracking noises, vertigo on assuming the erect posture, intolerance of light and sound, wakefulness, starting during sleep, waking hurried and alarmed, faintness, feeling of sinking, of impending dissolution, overcome by noise, disturbance, and thinking even, and delirium.

"*The heart* is in different cases affected with palpitation, fluttering, irregular and feeble action; there are beating and throbbings of the carotids, and sometimes even of the abdominal aorta; great rapidity and sometimes irregularity of the pulse; faintness or fainting; urgent demand for the smelling-bottle, fresh air, fanning, bathing of the temples; feeling of impending dissolution; incapability of bearing the erect position; and sometimes early fainting from V. S.

"*The respiration* is affected in different cases with panting, sighing, heaving, gasping, moaning, blowing, catching, with urgent demand for fresh air. There is sometimes great and alarming oppression about the chest. There is in some cases an irritative cough.

"*The stomach* is liable to become affected with irritability, sickness, retching, vomiting, hiccough and eructation; the bowels with constipation or diarrhœa, pain, flatus, distension, etc.

*Muscular system.*—"There are very frequently urgent restlessness, tossing about and jactitation. In some cases various spasmodic affections have occurred.

"*The seats of pain* are usually the head, the side, the illiac region, the loins, the uterus, and the abdomen generally. The pain of the illiac region and of the abdomen are often attended with much tenderness."

Dr. Hall impresses the idea that this affection will never bear blood-letting, although the local affections often led the practitioner to believe inflammation present. He does not attempt to explain the nature and pathology of the affection. The broken down and debilitated are its victims, and the greater the weakness the greater the susceptibility. *Males are sometimes attacked with this complaint.* The indication in the treatment is to restore the vital energies.

*Remarks.*—It is very evident from Dr. Hall's account of this affection, its causes, its subjects, its very varied and grave constitutional symptoms, with complete prostration of the vital powers, while seeming grave local inflammations are apt to set in and mislead in the treatment, that the "serious affection" described by him in 1820, in the British Journals, is identical with the "nursing sore mouth" affection first described in the Journals of this country in 1830 by Dr. Hale; at least it appears so to us, and the anomalous character of the cases being such as to prevent Dr. Hall from naming the disease, is strong confirmation of this. The identity of the two affections being conceded, we here establish another point of great importance in our researches, viz: that *males are sometimes subjects of the complaint.* This accords with our experience, although the idea has never been hinted in the Journals of this country. We have repeatedly seen men and boys subjects of the complaint, and also girls, as well as suckling women.—Hence we infer that pregnancy and lactation are causes of the affection only so far as they tend to impair the general health by confinement or want of proper exercise in the open air, improper restrictions in diet, a gloomy state of mind, exhausting drains by hemorrhage, etc.

The special attention of the reader is invited, in these cases of Dr. Hall, to the prominent symptoms, viz: lassitude, sinking, fainting, icteric pallor and anæmia, pain and tenderness of the abdomen, diarrhœa or constipation, aphthous ulcers and other local lesions, hemorrhagic associations, protracted course, getting well when the exciting cause was removed, as an exhausting menorrhagia or lactation, fatal tendency, sudden mode of death after some shock, as parturition, etc. We wish the reader also to notice the sudden development of the disease sometimes after a shock, as blood-letting, catharsis, or merely from the emotional shock of fear. All these are very instructive illustrations.

*Contributions of American Physicians.*—As before observed, Drs. Hale and Backus appear to have been the first to describe this affection in the United States, and an analysis of their contributions might, very properly, take the lead; but we prefer to place the most accurate account of the disease that has met our view first in order, and therefore give the substance of

*Dr. McGugin's Article.*—This valuable contribution appeared in the *Western Medico-Chirurgical Journal*, published at Keokuk, Iowa, October, 1851, and is styled "*Stomatitis in Pregnancy and during Lactation.*" In the outset, the affection is stated to be the ulcerative sore mouth consequent upon and occurring during pregnancy and nursing, particularly described by Drs. Hale and Backus. The idea is entertained that it is a disease peculiar to this country, rare on the sea-board, more common elsewhere. The doctor had met with several cases in two years' practice at Keokuk—says its pathology is not understood, and why it appears in certain localities, and whether or not caused by miasm, not known to the profession. Thinks local causes only act feebly in its production, that the real cause is in the system, for that the disease is relieved when the infant is removed from the breast—has found the scrofulous constitution most subject to it—thinks it arises from faulty nutrition, and that pregnancy, which favors excitement in the lymphatic system, somehow tends to develop it—that the glands of the mouth largely partake in the excitement, and in lax and strumous habits the feeble integrity of the tissues gives way to ulceration. Passing from the doctor's theories, we give his more valuable delineation of symptoms.

"The patient complains of burning heat, similar in sensation to that produced by hot fluids, when taken into the mouth. Food, when taken, even of the blandest kind, is swallowed with pain and difficulty, and that which is solid is masticated imperfectly and painfully. The lower lip is tumefied, and turns out and downward, and in the efforts to speak, the saliva, limpid and scalding, pours over it. There is pallor of the face, an anxious and painful expression of the countenance, and a crescentic dark circle below the eye.—The pulse is small and frequent. the skin dry, and the mind irritable and despondent. The mucous lining of the mouth is highly vascular and livid in color, the tongue red and often swollen, and early in the attack small granular elevations may be seen along its edges and tip, and still more highly vascular than even the surrounding mucous membrane. These points are highly sensitive, and much suffering is produced when the tongue touches the teeth or jaw. Very soon these show yellow vesicles on their tops, and in a short time these burst, leaving an ulcerated tip or depression, and rapidly, under the ulcerative process, extend themselves over the surface. They now multiply in number, and may be found within the lip, under the tongue, within the cheeks, and in the fauces. Now the suffering is great, for the surfaces of these ulcers are most sensitively endowed. They may extend down the œsophagus into the stomach, throughout the intestinal tube, into the posterior nares, down the trachea, along the bronchiæ, and finally involve the lungs in an irreparably diseased condition."

*Case.*—"Mrs. W———, of scrofulous predisposition, and advanced to the seventh month of pregnancy, had been laboring under stomatitis for three weeks before advice was taken. The tongue, the lips, the cavity of the mouth and fauces, were thickly covered with ulcerated patches. From the difficulty of swallowing, it was most manifest that it had proceeded downward along the lining of the œsophagus, and it was just as evident that the mucous coat of the stomach was also seriously involved. There was much suffering on deep pressure in the epigastric region, and food was rejected immediately upon swallowing it. The symptoms pointed to a diseased condition of the cardiac orifice. There was at this time some constipation of the bowels. After a time, from a change in the voice, together with a sense of tightness of the part, and stiffness of the muscles of the neck, it was evident that the larynx and trachea were suffering also. There was slight cough, with a muco-sanguineous expectoration, and upon retiring to bed, the semi-recumbent posture was chosen to favor inspiration, in which position the head was thrown back. There was slight dullness upon percussion over the entire thoracic surface. There was feeble respiratory murmur, owing to the thickened walls of the larynx, aided, doubtless, by the preternatural smallness of the chest.

"She had now arrived at the eighth month of her pregnancy, the previous month having been spent in the foregoing developments. The symptoms now assumed a more grave character; the cough was constant and harrassing; the sputa thick, tenacious and slightly sanguineous; wandering pains through the chest; respiration difficult at intervals; dullness over the entire thoracic surface, particularly manifest in the superior sternal and the right clavicular regions. There was bronchial respiration, but no vesicular murmur; irritability of the stomach so great as to reject food, drink or medicine. The dejections showed a large admixture of thick tenacious mucus, similar to that expectorated.

"About the middle of the eighth month the following were the symptoms: There had been a large discharge of pus in coughing; pectoriloquy in right infra-clavicular region; the ulceration had extended to the posterior nares, followed by alarming epistaxis, doubtless from the destruction of a vessel in the progress of ulceration; the irritability of the stomach continues as before; colliquative diarrhœa and hectic fever; cough persistent; sputa purulent and muco-purulent.

"Her confinement, which was now close at hand, was looked forward to as an event which would close her sufferings. All the symptoms continued in an exalted form up to this period, when uterine contraction came on, and her labor was concluded in two hours from the first evidence of uterine effort; was easy, and followed by but little loss in discharges. She, however, began to sink rapidly, and in eight hours from the delivery of the child she died. The child was less than the average, but appeared healthy. In a few days, however, as I learned, it sank rapidly with similar symptoms to those of the mother.

"The treatment was such as would naturally suggest itself; but the irritability of the stomach opposed a serious difficulty to the prosecution of any treatment, or a fair trial of any remedial agents internally."

In commenting on this case, Dr. M'Gugin notes the rapid development and extent of the local lesions, particularly phthisis, which is ordinarily hushed in pregnancy, but here it was developed.

Where the disease comes on after delivery or during lactation, he says, "it is attended with prostration, and even before the attack there is a sense of sinking and depression." Thinks the drain upon the system in the secretion of milk favors the rapid development of the disease, and therefore the infant should be separated from the mother before she is permitted to sink into hopeless anæmia. Suggests that animal chemistry may, by analysis of the milk, yet throw light on the pathology of the disease. Illustrates the imminent hazard that sometimes presents—the mother laboring under this affection, and the infant in the critical period of dentition—to wean may be death to the child, and not to wean death to the mother. Such a case came under the doctor's care the summer previous, and caused him much anxiety, but he saved both mother and child. The cure of the mother was ascribed to hydriodate of potash, a nutritious diet of broths, and porter as a drink. Several subsequent cases, he says, yielded to the same course of treatment; one especially, where, in a prior attack, weaning the child had to be resorted to in order to save the mother. Dr. M'Gugin says he has no confidence in any other remedy. The hydriodate of potash in solution also recommended as a gargle, and iodine to be added where the ulcers are "dark and ill-conditioned." Cinchona, porter, nourishing jellies and soups, and warm saline baths. If these means fail, wean the child.

*Remarks.*—Dr. M'Gugin's description of the disease and case reported illustrate the symptoms, progress, fatal tendency and sudden mode of death after a shock to the system, so accurately, that nothing is wanting but the anatomical characters to render his contribution complete. To be sure he infers what, no doubt, an autopsy in the case would have revealed, viz: ulcerations or lesions of the epithelium throughout the gastro-pulmonary branches of the mucous membrane—that is, in spots and patches. The sero-sanguineous salivation, alarming epistaxis, and development of tuberculosis, justify this conclusion.

We have witnessed several instances of *sudden and unexpected death* after delivery in this affection, as noticed by Dr. M'Gugin and Dr. Marshall Hall, and shall speak more at length of this feature of the disease in our analysis of cases. Suffice it that we invite especial attention to it in this connection, as also to the *hemorrhagic tendency* spoken of by Drs. M'Gugin and Hall.—The reader should also bear in mind the *prostration and sinking*, spoken of by Dr. M'Gugin before the disease comes on—that is, before the mouth becomes sore. This is doubtless the "forming or fixing stage" of the affection spoken of by Dr. Judkins. The local lesions, then, are secondary, according to these contributors, which accords with our experience. Here then we establish another important point in our researches, viz: that the local lesions of the mouth, fauces, stomach and bowels, posterior nares, and bronchial membranes, are consequent upon the general or constitutional affection. One other symptom, or rather objective sign, noticed by Dr. M'Gugin, is worthy of special attention; it is the highly vascular and *livid color* of the mucous lining of the mouth, spoken of. The papillary blisters or yellow vesicles that form on the sides of the tongue, and spread to all parts of the mouth and fauces, stomach and bowels, etc., are spoken of by others, as are constipation and diarrhœa. We wish the reader also to take note that a *salt of potash* is Dr. M'Gugin's main remedy; not that he was the first to call attention to the efficacy of a salt of potash or soda in this disease, but because he bears testimony to this fact.—In the dietary he prescribes, viz: *nourishing jellies and soups*, aided by porter, cinchona, and warm saline baths, he agrees well with other contributors. Calling attention to one other circumstance, viz: the sinking and death of the infant "with similar symptoms to those of the mother," we will close our comments on this very instructive contribution.

*The Contributions of Drs. Hale and Backus* are made the basis of the descriptions of this affection to be found in *Stokes and Bell's Practice*, second edition, page 54; and also in *Wood's Practice*, third edition, vol. 1, page 500. Both of these standard authors, Bell and Wood, have taken it for granted that this is a new form of disease, without ever having seen it, or had an opportunity of investigating its character! Both reflect the opinions of Drs. Hale and Backus, that the disease is at first a local affection or ulceration of the mouth, extending by degrees to the fauces, stomach, and bowels, and thus secondarily involving the constitution, and breaking down the general health; and both follow the dogma inculcated by these and most other contributors, that the disease is peculiar to suckling women, though it may possibly occur in the latter months of pregnancy. According to these authors, Dr. Hale's first communication on the subject is to be found in the *Medical Communications of the Massachusetts Medical Society*, vol. v., 1830 ; and his second, in the *American Journal of the Medical Sciences*, April, 1842. Dr. Backus' article in the latter, January No. 1841. We have not seen the original contributions, but the substance of them, as set forth in the articles styled " *Stomatitis Nutricum*," by Dr. Bell ; and " *Sore Mouth of Nursing Women*," by Dr. Wood, which are the sources of this analysis of Drs. Hale and Backus' views, and which for reasons that will be obvious shortly, we feel it to be particularly incumbent on us to carefully reflect.

According to Bell and Wood's works on Practice, then, the incursion of the disease is sometimes sudden, and the local affection of the mouth is characterized by loss of taste, scalding sensations, patches of painful pimples on the sides of the tongue and mouth, which after a time ulcerate, and produce very painful sores, with hard elevated edges, and an inflamed circle around them The inflammation, as the disease progresses, extends over the mouth by means of successive crops or patches of these papillæ ; the surfaces of the mouth become exquisitely tender ; the taking of food and drinks causes much pain ; and a copious salivation sets in. This *local* disease, as it is considered, is not at first attended by febrile symptoms, loss of appetite, or furred tongue, but on the contrary the appetite is good througuout the course of the disease, and the tongue is red and smooth ; but if the disease be not arrested, ulceration extends to the fauces, œsophagus, stomach, and bowels, and then great intestinal irritation and severe constitutional symptoms supervene, with diarrhœa, debility, emaciation, etc., which overwhelm the patient and often end in death. This gives a condensed outline of the symptoms as recorded in the standard works of Drs. Bell and Wood, professedly drawn from the contributions of Drs. Hale and Backus. Dr. Hale, it is stated, has seen considerable loss of the substance of the tongue by sloughs ; and Dr. Backus has noticed so sudden an attack that, " in three hour's time after seeing your patient in health, you may find her with a scalded tongue and fauces, and unable to converse or take food." [!]

The cause of the disease is ascribed to some unknown baneful influence, exerted on the system by nursing. [!]

The subjects are wholly women in the suckling condition ; or if pregnant women are attacked, they are those who had had the disease previously while suckling, and had established a predisposition to it. It is very apt to recur in subsequent nursings if a woman has once had it.

The constitutions most liable to it are the leuco-phlegmatic and dyspeptic, which are habitually costive ; but others, even the most robust, are sometimes its victims. It appears to be much more prevalent in some localities than others.

The prognosis is generally favorable, if the constitutional symptoms of exhaustion have not run too long; and it is chiefly where there is a predisposition to phthisis that alarm need be felt, and the child weaned. Notwithstanding the wasting and weakness of the mother, the secretion of milk holds out, and the child continues vigorous and healthy.

The treatment is rested mainly on tonics, laxatives, *lemon juice and bicarbonate of potash* in effervescing draughts, *tartaric acid* in small beer, and porter, with nourishing diet; at least this is Dr. Hale's treatment, and very little value is attached by him to local treatment. Dr. Backus recommends chalybeates combined with laxatives. The nitrate of silver is recommended as a local application. Weaning the child is thought to be an effectual measure.

*Remarks.*—The above is a faithful abstract of the accounts given of this affection, by Drs. Bell and Wood, drawn from the contributions of Drs. Hale and Backus; and no one, we presume, will question the identity of the disease with that described by Drs. M'Gugin, J. P. Hall, Marshall Hall, Ellsworth, and Judkins.—The several descriptions all comport in the main. There is nothing particularly important in the descriptions of the affection as drawn from Bell and Wood, over and above that of others, on which we can rest a point, save in the treatment, viz: that *lemon juice*, *tartaric acid*, and *a salt of potash* are effectual remedies.

To post up the several points made in our researches, by way of keeping the mind refreshed, we find we have made the following, viz: 1. The nursing sore mouth is not an affection peculiar to nursing women. 2. It manifests itself epidemically after cold protracted winters. 3. Males are sometimes its subjects. 4. The local lesions are secondary. 5. The vegetable acids and salts of potash are effectual remedies. These points we think are clearly established, to say nothing of the balance of the testimony, all tending in the same direction, and, as we think, conclusively proving that this anomalous affection, which has been stumbled over by the profession in Europe and America for the last thirty years, is nothing more nor less than LAND SCURVY. This has been our conviction for many years, since 1835, when we first encountered it, and met it successfully with anti-scorbutics. In 1851 we reported sundry cases of it successfully treated on this plan, in the *New York Journal of Medicine*, and presumed it was only necessary to call attention to its real and true nature to have the matter fully appreciated. We there remarked as follows: "These cases are reported because of their practical bearing. Marvellous accounts of this nondescript disease, called 'nursing sore mouth,' appear from time to time in the journals; and why some one has not set its nature and pathology to rights, who is in the habit of contributing to, and fond of appearing in the journals, I am at a loss to understand." But it seems "we reckoned without our host," for Dr. Wood still adheres to opposite conclusions, which are being widely disseminated. He closes his article on "*Sore Mouth of Nursing Women*," with an allusion to our views as follows: "Dr. M. L. Knapp, formerly Professor of Materia Medica in the University of Iowa, considers this disease as essentially scorbutic; and has treated cases on this principle successfully, which have come under his notice; but the affection, as described by Drs. Hale and Backus, has not the peculiar features of scurvy, and differs probably from that noticed by Dr. Knapp."

Now our object in elaborating this subject is to arrive at the truth. Dr. Wood is a prominent author, his opinions have deservedly much weight, and, as we are at issue on a question of fact, as to whether or not the affection described by Drs. Hale and Backus presents the peculiar features of scurvy, we invite a careful attention to the subject. If a scalded, driveling sore mouth in female subjects, exhausted and prostrated by breeding and nursing, displaying increasing patches of ulceration of the mouth, tongue, fauces, and extending to the bowels and bringing on diarrhœa, profuse salivation and complete prostration of the system, development of grave and complex constitutional symptoms and local lesions—if these phenomena do not present the leading "peculiar features" of land scurvy, we ask what symptoms do? and if lemon-juice, tartaric acid, and a salt of potash prove effectual remedies, what then? We are not ambitious to "shiver a lance" with Dr. Wood; but nevertheless, as our views are crowded to the side of error by him, in the diagnosis of a disease in which we have had much experience at the bed-side and he none, we owe it to medical science and humanity, to endeavor to right ourself, and stay the promulgation from so high authority of so absurd a medical philosophy as teaches that this is a *new disease;* that it is *peculiar to nursing women;* that it is caused by some *unknown baneful influence exerted on the system by nursing, etc.. etc.;* and that denies that *soreness of the mouth in a suckling woman, anæmia, prostration, salivation and diarrhœa*, present the "peculiar features" of scurvy; and where, in the same breath, it discloses unwittingly the reason why *lemon-juice, tartaric acid and a salt of potash* are reliable remedies!

*Dr. Channing's Article.*—In the *New England Quarterly Journal of Medicine and Surgery*, for October, 1842, is an article on this affection, with cases, which is epitomized in the *Maryland Medical and Surgical Journal* for December of the same year, as follows:—*Notes on Anhæmia, principally in its Connections with the Puerperal State*, by W. CHANNING, M.

D. The cases detailed in this article are quite interesting, and seem to demonstrate the existence of some pathological condition other than the loss of blood, producing the condition which Dr. C. thinks is improperly called *anhæmia*. He ventures to suggest, and sustains his suggestions with great plausibility, that this pathological condition consists, if not entirely, at least in great part, in the subversion of the functions of the capillary system, by which the blood passes from the arteries to the veins, without undergoing its usual changes. The symptoms of this condition are a brilliant whiteness, smoothness, roundness, dryness and warmth of the surface every where; the blanched lips, mouth, tongue; the scarcity of external or subcutaneous veins, and the bright pink color of their contents, with the want of the roundness in these vessels, which results from fullness, various noises in the head, the mind in various states, but generally having a serene anticipation of death.—There is tumultuous action of the heart. Dr. Channing, sustained by his cases, infers a close connection between the puerperal state and this morbid condition, and in such a connection the disease is most fatal. The cause of the disease being so obscure as it is, the treatment is necessarily undefined, and can only answer obvious indications. Dr. C. suggests as an inquiry, what might be the effect of transfusion?

*Remarks.*—The 'puerperal anæmia' of Dr. Channing is evidently the 'serious affection' of Dr. Hall, and the 'nursing sore mouth' of other contributors. Doubtless also it is the 'hydræmia gravidarum' and 'endangium out of order' of Dr. Meigs, and the 'leucocythemia' of Dr. Bennett. There is something behind the scene in the pathology of all these watery blooded cases, with a powerless fiber, palpitating heart, panting respiration, and a brain looking with serene anticipations on death, that stamp them as more than simply *anæmia*, or loss of blood, as Dr. Channing says, and we fully agree with him that the affection is improperly called anæmia, it is the scorbutic diathesis developed in various degrees and in various ways, and sometimes in delicate females by improper restrictions in diet under medical direction. The materials for healthy blood have been withheld from the dietary, or the organs of digestion so deranged that nutrition and assimilation have become starved of their rights. It appears idle to us to look for the cause of the difficulty in some unknown, mysterious disturbance of the functions of the capillaries, as Dr. Channing suggests, or in the endangium as Dr. Meigs conceives, or in the spleen as Dr. Bennett argues. No doubt the capillaries, the blood membrane, and the spleen are all at fault, and suffer from impoverished blood. All the solids are equally as hydræmic, or leucocythemic, as the blood itself; hence the softening of glandular structures, and local lesions of the tissues. Where the causes and the co-operating causes are acting powerfully, as in a delicate breeding woman who has suffered in the earlier months of her pregnancy from morning sickness, and in the latter months from heart-burn, and who has been dieted on tea and toast, ulcers of the mouth should break out before delivery; but under more favorable circumstances there may be no local lesions, and yet very marked constitutional derangement. We have met with cases of years' standing where there were no ulcers of the mouth, only a lividity of its tissues, without salivation, even; and we have known cases of the affection to terminate fatally where soreness of the mouth was not complained of at all.

This leucocythemial, or white cell blood diathesis, is particularly prevalent in malarious districts. We have seen much of it under such circumstances in both sexes. It is much more frequently met with, however, in females, from the chlorotic girl to the suckling mother, than in males, as the victims of land scurvy are "principally women." (*Good.*) Iron alone is not a sufficient remedy, though we agree with Dr. Meigs (Letters to his Class *On Woman and her Diseases*,) that it is a good one; but all the elements of a healthy nutrition, as furnished in the "good rich diet" advised by Dr. Judkins, and the "nourishing jellies and soups" recommended by Dr. McGugin, together with the acidulated drinks of Dr. Hale, must be judiciously brought to bear upon these cases, aided by tonics, and a correct hygiene; which being found effectual, proves our views of the nature of the malady correct.

Dr. Channing infers a connection between the puerperal state and this morbid condition, in which connection the disease is most fatal. Others have inferred its connection only with the later period of lactation; but we have seen that it is also associated with gestation, and furthermore, that it is often the inheritance of infancy. The majority of infants at the breasts of mothers laboring under it, imbibe it. This we have verified over and over again, during the last twenty years, by the success of the lemon-juice treatment. We have rescued hundreds of puny, suckling infants, covered with indolent boles, or wasting under diarrhœa, simply by the administration of lemon-juice to the mothers, and throwing away the blue pill mass, etc., with which they were being drugged. Now the records of scorbutus show that infants imbibe the diathesis from the impoverished materials afforded by the milk of mothers laboring under it. And who so blind as not to see the identity? But let us briefly trace this infantile inheritance from nursing sore mouth or scorbutic mothers. If the infants live to be weaned, thousands upon thousands of them perish of cholera infantum during their second year. If some of them reach puberty, the vis vitæ is too feeble, and green-sickness sets in, and ends in the local lesion of tuberculosis. If chlorosis be averted by chalybeates and a proper hygiene, still the leuco-phlegmatic constitution is formed in the girl, as her lax solids and palid countenance, lucorrhœa, falling of the womb, and tendency to local lesions of the os, import; and when this victim becomes a mother, nursing sore mouth sets in as a matter of course. Our philosophy, then, goes further back than simply to note that the leuco-phlegmatic constitution is the one for ever liable to nursing sore mouth; it explains the *cause* of this constitution, lifts the curtain and gives us a peep behind the scene.

*Dr. Shanks' Article.*—This article, *On Enpidemic Sore Mouth and Diarrhœa peculiar to Nursing Women*, by LEWIS SHANKS, M. D., of Memphis, Tennessee, we find epitomized in the December number of the *Maryland Medical and Surgical Journal*, December, 1842, drawn from the *American Journal of the Medical Sciences*, October, 1842, as follows: "In the treatment of this malady, when it occurs in the last months of gestation, as it sometimes does, Dr. S. relieves the excitement in the robust and plethoric by bleeding, followed by alteratives and laxatives, such as blue mass, calcined magnesia, and rhubarb in small doses; in those of feebler health, in whom there is little or no feverish excitement, he prescribes, as a tonic and alterative laxative a combination of blue mass, ipecac, carb. of iron, rhubarb and aloes in proportions to suit each case. Ipecac alone, in doses of from one-half to two grains, is a good remedy.

During nursing, when the disease becomes chronic and is attended with diarrhœa and emaciation, a course of alteratives and a rigid attention to diet are indispensable. In some bad cases a solution of arsenic and corrosive sublimate, containing of each a sixteenth of a grain for a dose, given two or three times a day, with a diet and drink of soda with barley water, or of wine and water and milk, has succeeded better with Dr. S. than any other course he has tried. As a wash for the mouth, the infusion of sanguinaria is recommended by him. Weaning the child is indispensable in grave cases attended with much emaciation and nervous irritation. In a description of this disease as it occurred at Rochester, N. Y., Dr. Backus says, the onset is often sudden and the bowels always constipated; and the most successful treatment is with alteratives and laxatives combined; but at Memphis, Tennessee, it comes on gradually and in its well-marked chronic form, which never occurs except during lactation; the constant diarrhœa excludes the use of purgatives or laxatives. In the same city, and in the level alluvial country near it, constipation is rare either in health or disease.

*Dr. Taylor's Article.*—We find an epitome of this also in the *Maryland Medical and Surgical Journal*, March, 1843, drawn from the January number, same year, of the *American Journal of Medical Sciences*. It is entitled *Remarks on a Species of Sore Mouth peculiar to Nursing Women;* by B. W. TAYLOR, of Monticello, Va. "After having tried various tonics, vegetable and mineral, and laxatives, with only partial success, Dr. T. has found that equal parts of sulphur and cream of tartar in broken doses, to keep the bowels open, constitute the best treatment as regards internal remedies. It appears to have almost a specific influence over this disease. The best external application he thinks is borax. He has also derived great benefit from a weak solution of nitrate of silver. In cases attended with considerable exhaustion, the sulphur and cream of tartar should only be used to the extent of obviating costiveness, if it exist; and tonics, such as iron, cinchona, and elixir vitriol should be given. Porter is also advised. Should the case be complicated with diarrhœa, opiates should be given, with mucilaginous drinks and farinaceous diet. In cases that prove refractory, wean the child, when a speedy cure will take place."

*Remarks.*—This article furnishes further proofs of the efficacy of the salts of potash and soda in this disease.

*Dr. Ware's Article.*—In the *American Journal of Medical Sciences*, 1849, is a brief article on "*Nursing Sore Mouth*, by J. YALE WARE, M. D., of Massachusetts," stating that the affection is rapidly on the increase in that locality. No symptoms or cases are given, but Griffith's myrr. mixture is recommended as an infallible remedy. Nitrate of silver as a gargle is also advised, of the strength of two grains to an ounce of water, and a teaspoonful to be swallowed three times a day if soreness extends to the stomach. It is believed if this course is pursued weaning need not be resorted to.

*Dr. Holt's Article.*—A brief article appeared in the *New York Journal of Medicine*, in May, 1848, from the pen of HENRY D. HOLT, M. D., of New York, recommending the hydriodate of potash in five-grain doses three times a day, given in the compound decoction of sarsaparilla. This had proved effectual in sundry obstinate cases. The doctor says, "without propounding any theory of the pathology of the disease, or modus operandi of the medicine, I feel persuaded that the one is as near being a specific for the other as can well be conceived."

*Dr. King's Article.*—This article of Prof. John King, M. D., of Cincinnati, appeared in the *Eclec. Med. Jour.*, April, 1852, and although it gives a similar account of the disease to that which may be found in Bell or Wood, presents nothing particularly new in the history or treatment, except that it is stated there is always a dry, inactive state of the skin, for which alkaline washes and the spirit vapor bath are recommended. And we take leave to add, that the skin is always *scurfy*, oftentimes sprinkled with *petechiæ*, and in some instances of extreme prostration we have seen *vibices*.

Thus much for the history and literature of the affection, embodying the present state of medical knowledge on the subject.—Some few other papers giving an account of the affection have appeared, we believe; but, so far as we have been able to learn, the disease has never been suspected of being of scorbutic character by any contributor save ourself. We are not able to say what the impressions may now be on the mind of the profession, after an examination of the literature of the affection with the key of explanation offered by us, nor are we anxious at all about the matter further than the interests of science and humanity are concerned. Indeed we would rather regret to have proselyted many to our views in this early stage of our inquiry, lest interest in the subject flag, and would rather invite a suspension of opinion until we shall have pushed our enquiry through its several chapters. A question in practical medicine as important as this, deserves to be well and carefully considered, and all opinions sifted before final conclusions be drawn. This chapter is but preparatory—the collated views of others. If after our researches shall have been completed, it turns out to be the general sense of the profession that the nursing sore mouth affection is a new disease, then let it be retained in Wood, and in Stokes and Bell, and inserted in other standard authors; but if it be proved to be *scurvy*, let us study that disease instead, which we think, has been culpably overlooked of late years.

Doubtless the same liability exists in the human constitution, to take on the scorbutic diathesis, that has existed since the days of Hippocrates, by whom it was first described, and that existed in all time before, and that will always exist to the end of time, under a faulty alimentation or a meagre supply of proper materials for forming healthy blood, perverted digestion, and obstructed æration.

This faulty condition, no doubt, obtains to a very considerable extent in the present state of society and modes of life, even under our improved notions of the etiology of scurvy and the more general attention paid now-a-days to gardening and fruit culture.—The poor inhabitants of cities, who are compelled by necessity to the daily infraction of the laws of a healthy dietary, are thereby rendered more or less scorbutic unquestionably, a standing cause of the aggravation of all their diseases, and of the vast amount of infantile mortality in our large cities; and we shall show conclusively, before we finish these researches, that the rich and highborn are frequently rendered the victims of scorbutus by mistaken notions as to what constitutes a proper and wholesome dietary; in other words, that restrictions in diet together with sedentary habits lay the scorbutic diathesis in the symptoms of many a breeding woman in high life.

